# TEACHING MUST GO ON: flexibility and advantages of peer assisted learning during the COVID-19 pandemic for undergraduate medical ultrasound education – perspective from the “sonoBYstudents” ultrasound group

**DOI:** 10.3205/zma001401

**Published:** 2021-01-28

**Authors:** Nasenien Nourkami-Tutdibi, M. Hofer, M. Zemlin, H. Abdul-Khaliq, E. Tutdibi

**Affiliations:** 1 Saarland University Medical Center, Hospital for General Pediatrics and Neonatology, Homburg/Saar, Germany; 2 Saarland University Medical Center, Hospital for General Pediatrics and Neonatology, sonoBYstudents, Homburg/Saar, Germany; 3 University Spital Bern, Institute for Diagnostic, Interventional and Pediatric Radiology, Inselspital, Bern, Switzerland; 4 Saarland University Medical Center, Hospital for Pediatric Cardiology, Homburg/Saar, Germany

**Keywords:** ultrasound, undergraduate medical education, peer teaching, peer assisted learning, digital learning, COVID-19 pandemic

## Abstract

**Background: **Facing the global COVID-19 pandemic University teaching has been digitalized and German medical faculties took great effort to offer curricular contents online as they agreed that semesters during pandemic should not be suspended. Skill training is an essential part of medical education and cannot be fully digitalized nor should it be omitted. The pandemic demonstrates that skills like ultrasound are essential when treating critical ill patients. Medical faculties use peer assisted learning (PAL) concepts to teach skills, like ultrasound through specially trained student tutors.

**Aim: **Here, we would like to share our experiences and elaborate how ultrasound teaching can be safely performed during the pandemic with an emphasis on adjustment of an existing PAL teaching concept.

**Method:** At the hospital of Saarland University, we implemented a PAL teaching concept for abdominal, including emergency, ultrasound, and echocardiography, called “sonoBYstudents” to teach sonography to undergraduate medical students. Students are generally taught in small groups of 5 people in 90min sessions over a time of 8 weeks with an objective structured clinical exam (OSCE) at the end of the course program. Each semester nearly 50 students are taught in abdominal and emergency ultrasound and 30 students in echocardiography. Over five years, more than 600 students have been taught with at least 30 students being trained as student tutors. Given the pandemic, course size, course interval and total course time and total course time were adapted to the hygienic precautions.

**Results:** 45 and 30 students were taught in abdominal ultrasound and echocardiography respectively achieving their learning goals measured via OSCE at the end of the courses. OSCE results were the same when compared to previous semesters.

**Conclusion: **PAL as a teaching concept lives out of sustained educational strategies like practical and didactical trainings and an ongoing recruitment of new student tutors. Suspending PAL and its skill teaching would require starting from the beginning which is a time and cost consuming process. With sonoBYstudents we were able to demonstrate that an existing PAL concept can, with some effort, be adjusted to changing teaching circumstances. Apart from this ultrasound is a non-omittable part of medical skill training with easily appliable hygienic precautions during teaching sessions.

## Zusammenfassung

**Hintergrund: **Die globale COVID-19-Pandemie führte zu einer raschen Digitalisierung der universitären Lehre. Innerhalb der medizinischen Fakultäten Deutschlands herrschte der Konsens, dass während der Pandemie kein Semester ausgesetzt werden soll. Daher wurden mit viel Mühe, weite Teile der Lehrinhalte online angeboten. Das Unterrichten von praktischen Fertigkeiten ist ein wesentlicher Bestandteil der medizinischen Ausbildung und kann nicht digitalisiert, jedoch auch nicht ausgelassen werden. Die Pandemie zeigt, dass praktische Fertigkeiten wie Ultraschall bei der Behandlung kritisch kranker Patienten unerlässlich sind. Die medizinischen Fakultäten in Deutschland nutzen daher Peer-Assisted-Learning (PAL) Konzepte, als modernes Lehrmodel, um solche praktischen Fertigkeiten, wie Ultraschall, durch speziell ausgebildete studentische Tutoren zu vermitteln.

**Ziel: **Wir möchten unsere Erfahrung teilen und aufzeigen, wie der studentische Ultraschallunterricht während der Pandemie sicher durchgeführt werden kann, wobei der Schwerpunkt auf der Anpassung eines bestehenden PAL-Lehrkonzepts liegt.

**Methode: **An der medizinischen Fakultät der Universität des Saarlandes, führten wir vor 5 Jahren ein PAL-Lehrkonzept, genannt „sonoBYstudents“, ein um Medizinstudenten in der Abdomen-, einschließlich Notfall-Sonographie und Echokardiographie zu unterrichten. Die Studenten werden in Kleingruppen à 5 Personen in 90-minütigen praktischen Seminaren über einen Zeitraum von 8 Wochen unterrichtet. Der Lernerfolg wird durch eine validierte OSCE (objective structured clinical examination) am Ende des Kursprogramms überprüft. Jedes Semester werden ca. 60 Studenten in der Abdomen- und Notfall-Sonographie und 30 Studenten in der Echokardiographie unterrichtet. In fünf Jahren konnten mehr als 600 Studenten unterrichtet sowie mehr als 30 Studenten als studentische Tutoren ausgebildet. Aktuell wurden angesichts der Pandemie Kursgröße, Kursintervall und Gesamtkurszeit, entsprechend einem Hygienekonzept, angepasst.

**Ergebnisse: **45 bzw. 30 Studenten wurden in der Abdomensonographie und in der Echokardiographie unterrichtet und erreichten am Ende der Kurse ihre über die OSCE gemessenen Lernziele. Die Ergebnisse der OSCE zeigten keinen signifikanten Unterschied im Vergleich zu den Vor-Semestern.

**Schlussfolgerung:** PAL als Lehrkonzept lebt von nachhaltigen Ausbildungsstrategien wie praktischen und didaktischen Schulungen und einer kontinuierlichen Rekrutierung neuer studentischer Tutoren. Das Pausieren eines bestehenden PAL-Konzeptes und seiner Kompetenzvermittlung würde eine beinahe neue Implementierung nach Pausierung erfordern, was ein zeit- und kostenaufwändiger Prozess ist. Mit sonoBYstudents konnten wir zeigen, dass ein bestehendes PAL-Konzept mit etwas Aufwand an sich ändernde Unterrichtsbedingungen angepasst werden kann, ohne den Lernerfolg der Veranstaltung zu mindern. Letztlich ist die Sonographie unverzichtbarer Teil der medizinischen Ausbildung und eine wichtige medizinische Fertigkeit. Wir konnten zeigen, dass die Ultraschallausbildung im Medizinstudium mit für jeden leicht anwendbaren und nachvollziehbaren hygienischen Vorkehrungen während des Kleingruppenunterrichts fortgeführt werden kann.

## Introduction

Reaching end of 2020, the worldwide COVID-19 pandemic has already changed crucial aspects of our society. Many clinicians at university hospitals determined how to adjust educational activities to meet the new circumstances. Skill training is an essential and non-omittable part during medical education and cannot be fully digitalized. The pandemic revealed how important essential skills as point-of-care ultrasound (POCUS) and lung ultrasound are in treating critically ill patients [1]. While clinicians are faced to keep up with patients care, peer assisted learning (PAL) concepts are of advantage in regard of their flexibility and versatility especially when relying on an amount of specially trained students tutors. Many German medical faculties successfully established PAL for undergraduate medical education within their skills lab [2], [3]. Didactical and practical skilled trained student tutors are able to teach advanced medical skills like abdominal ultrasound or echocardiography [4], [5], [6]. Here, we would like to outline the importance and advantages of ultrasound teaching to undergraduate medical students and how an existing peer teaching concept with little adjustments can meet the requirements of new teaching circumstances. 

## Method

The medical faculty of Saarland University, Children Hospital Homburg/Saar, developed and implemented a PAL concept called “sonoBYstudents” to teach ultrasound to medical students [7]. For more than 5 years, abdominal and emergency ultrasound as well as echocardiography is taught to approximately 60 and 30 students respectively per semester with more than 30 student tutors being trained since then. Each semester new students are recruited and trained to become future tutors performing a special skill and didactical training before teaching their own ultrasound course. The course curriculum is based on the concept developed by Matthias Hofer [7], [8] including free online lectures [9]. Our ultrasound courses are available as an elective subject and are not yet part of the mandatory curriculum. The courses are well established and known among students and faculty staff members and before the pandemic, held as small group trainings with 5 persons and one supervising tutor weekly for 90min with different topics over 8 weeks. Generally, 10-12 specially trained tutors are employed every semester with new tutors being recruited every semester. During the pandemic, we adjusted the course format to cause as less personal frequency as possible. Hygienic restrictions required reduction of course size to 4 persons and courses were held as half day compact courses on two days with 6h sessions/day (total 12h on two days). Upfront all registered students were asked whether they would like to join the adjusted course as it was supposed to be more intense than the weekly based format. Facemasks were mandatory as well as regular hand washing, and disinfecting of transducers, equipment surfaces and hands during the whole course time. To limit presence time on campus and to decrease risk of infection, all courses were held in a time frame of 2 weeks with an OSCE at the end of the course. All students registered online and agreed to be contacted in case of infection. A hygienic concept had to be submitted to the deanery before courses were held. 

## Results

45 and 30 students attended the abdominal and echocardiography courses, respectively. Teaching goal was achieved and the acceptance for the adapted course format was high as feedback was throughout positive. OSCE results were comparable to the previous semesters despite the adapted course format. 

## Discussion

Skill training, like ultrasound teaching is an important part of medical education and should not be omitted. Here we demonstrate how an existing peer teaching concept for ultrasound education can be adjusted to pandemic requirements. Teaching goals were reached and satisfaction among students was high. Independent of the current pandemic, accompanying online lectures are a main pillar in the preparation and learning process of advanced skills like ultrasound and should not be an “add-on” or “nice to have”-option. Regarding safety, skill training courses, like ultrasound teaching, should take place in rooms apart from patient care such as skills labs to maintain hygienic restrictions and to ensure student’s safety. During pandemic with online relied lectures PAL increases student’s autonomy being able to create and contribute to their own education and curricula. Student tutors were highly motivated to continue teaching as in times of online lecturing they are offering crucial skill training to fellow tutors. The latter leads to a student empowerment as they contribute to their own education with an awareness to be an active and full part of curricular development and academic teaching. Like any other skill, teaching skills, need not only to be trained, yet to be performed on a regular basis in a real teaching setting as all simulations and didactical training, as important as they are, cannot replace the real life setting. Maintenance of well-established PAL concepts is important for ongoing education of medical students and their student tutors running their course as well as the recruitment and teaching of new student tutors. PAL courses rely on a continuity from one generation of student tutors to the other. Suspending PAL courses would interrupt this tradition, requiring a start from the beginning, which is a time and cost intensive procedure. Moreover, every medical faculty has the teaching assignment to teach advanced practical skills, like ultrasound which cannot be fully realized despite excellent digital learning concepts. 

## Conclusion

Ultrasound teaching remains an important part of medical skill teaching and should be prioritized with all efforts. Hygienic precautions can easily be applied during ultrasound teaching sessions. An established PAL concept with highly motivated and well-trained student tutors is a realistic and feasible approach to sustain ultrasound skill training during medical education even when facing a global pandemic. A limitation of the adapted course concept might probably be the fact that long term retention is better when skills are learned and repeatedly trained over a longer time frame [7]. Therefore, we aim to continue ultrasound teaching during pandemic in small groups but we will be teaching weekly over a timeframe of 8 weeks again as feedback from evaluations demonstrated that the content within two half days was, even if feasible, felt to be too dense for many students. 

## Competing interests

The authors declare that they have no competing interests. 
